# Influence of laboratory and radiographic parameters on the clinical presentation and outcome of surgically treated patients with primary brain abscesses

**DOI:** 10.1007/s00701-025-06559-8

**Published:** 2025-05-15

**Authors:** Adrian Liebert, Thomas Eibl, Dimitri Lukin, Ralph Bertram, Joerg Steinmann, Karl-Michael Schebesch, Leonard Ritter

**Affiliations:** 1 Department of Neurosurgery, Paracelsus Medical University, Nuremberg General Hospital, Breslauer Straße 201, 90471 Nuremberg, Bavaria Germany; 2 Institute of Clinical Microbiology, Infectious Diseases and Infection Control, Paracelsus Medical University, Nuremberg General Hospital, Nuremberg, Germany

**Keywords:** Abscess, Surgery, Outcome, Risk factors, Intracranial

## Abstract

**Background:**

Brain abscesses can lead to severe clinical outcomes, including death. Most studies on brain abscesses focus either on patient cohorts including postsurgical/posttraumatic abscesses. This study aimed to assess the clinical presentation and postoperative outcomes in a homogeneous group of patients with primary, pyogenic brain abscesses who underwent surgical treatment.

**Methods:**

We retrospectively analyzed consecutive patients with pyogenic brain abscesses treated surgically at our center from 2008 to 2023. The primary endpoint was a modified Rankin Scale (mRS) score of ≥ 3 at discharge. Secondary endpoint was preoperative clinical status (mRS ≥ 3). We statistically correlated clinical, radiographic, and microbiological parameters with these endpoints.

**Results:**

A total of 60 patients (36.7% female) with a mean age of 48.5 ± 20.8 years were included in this study. Six patients (10.0%) had an unfavorable postoperative outcome, including two deaths (3.3%). Significant risk factors for poor outcomes included preoperative disturbance of consciousness (DOC) (*p* = 0.012) and elevated preoperative C-reactive protein (CRP) levels (*p* = 0.002). Larger abscess volume (37.4 mL vs. 16.1 mL, *p* = 0.065) and shorter mean distance to the ventricles (3 mm vs. 11.42 mm, *p* = 0.086) trended toward significance. The length of intensive care unit (ICU) stay was significantly longer for patients with unfavorable outcomes (*p* = 0.001). Upon admission, eighteen patients (30.0%) had an mRS score of ≥ 3. Elevated leukocyte count was identified as a significant risk factor for poor preoperative status (*p* = 0.007). Median clinical performance, measured by mRS, improved throughout the treatment course and during follow-up from 2 to 0.

**Conclusions:**

Preoperative DOC and elevated CRP levels were identified as predictors of unfavorable outcomes. Elevated leukocyte count was a predictor for poor preoperative status.

## Introduction

A brain abscess is a localized intracranial infection that can result from either the contiguous spread of pathogens or hematogenous dissemination, though in many cases, no identifiable cause is found [7, 16]. Brain abscesses typically develop in patients with predisposing factors, such as immunosuppressive diseases, systemic infections, treatment with immunosuppressive drugs, or disruption of the brain’s natural protective barriers [7, 11, 15].

Although the incidence of brain abscesses has increased from 0.4 to 0.8 per 100,000 adults over the past decades, they remain relatively rare [4]. Nevertheless, morbidity and mortality rates (ranging from 4.9% to 8.5%) remain high, despite significant advances in diagnostic and therapeutic approaches [1, 3, 5, 8–10, 20]. Early diagnosis and prompt initiation of appropriate treatment are critical to minimizing the risk of serious neurological complications [8, 18].

The introduction of antibiotics in the 1940 s offered an alternative treatment option for brain abscesses, alongside neurosurgery. However, neurosurgery remains a cornerstone of management for this life-threatening condition and is strongly recommended by the European Society of Clinical Microbiology and Infectious Diseases (ESCMID) as the preferred approach for most cases of brain abscesses [2, 6].

Most studies on brain abscesses examine mixed patient populations with varying treatment approaches (both conservative and surgical) or different etiologies (primary vs. secondary to surgery or trauma) [9, 11, 13, 22]. Among the few studies focusing exclusively on surgically and conservatively treated patients, the primary emphasis has often been on surgical outcomes, rather than on broader clinical outcomes [1].

The primary aim of this study is to identify risk factors associated with unfavorable outcomes in patients who underwent surgery for primary intracranial abscesses alongside antibiotic therapy.

## Materials and methods

### Study population

**Fig. 1:**
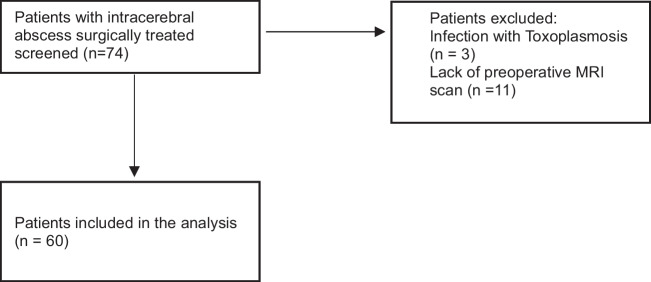
Flowchart of patient selection

Consecutive patients of all age groups who were treated surgically for a primary brain abscess at the Department of Neurosurgery, Paracelsus Medical University Nuremberg, between January 2008 and December 2023 were included in this retrospective analysis. Patients were excluded if they lacked preoperative MRI, received only conservative treatment, or had conditions such as tuberculosis, parasitic infections, or mycoses. A flowchart to visualize the patient selection can be found in Fig. 1.

### Study endpoints

The primary endpoint was an unfavorable outcome, defined as a postoperative modified Rankin Scale (mRS) score of ≥ 3 at discharge. The secondary endpoint was a poor preoperative status, also defined as an mRS score of ≥ 3. We also analyzed potential differences in outcomes based on the surgical technique used. Finally, follow-up data were collected to assess treatment success after hospital discharge.

### Surgical procedure and antibiotic treatment

Lesions were treated using one of two techniques: microsurgical craniotomy or frameless neuronavigation/frame-based stereotaxy aspiration. The choice of technique was determined on a case-by-case basis, depending on the size and location of the abscess. In all cases, pus was sent for microbiological analysis.

Antibiotic treatment was initiated only after obtaining a tissue sample, whenever possible. In all cases, empirical antibiotic therapy was started in accordance with current guidelines. Once a pathogen was identified, the regimen was adjusted in consultation with the microbiology department. Antibiotics were typically administered intravenously for four weeks postoperatively, followed by an oral regimen for an additional six weeks, continuing until the follow-up MRI. Patients remained in the neurosurgical department throughout their hospital stay.

### Data and imaging analysis

Medical records, surgical reports, pre- and postoperative laboratory results, and MRI scans were reviewed. Pre- and postoperative clinical status, including disturbance of consciousness (DOC), defined as a Glasgow Coma Scale (GCS) score of < 13, and comorbidities, were documented and assessed using the modified Rankin Scale (mRS) and the Charlson Comorbidity Index (CCI), respectively.

Regarding the medical records, we collected the presumed source of the abscess, including local spread (sinusitis, otitis), dental origin, entrance through skin lesions/wounds, and others. We also collected the patient’s immune status including rheumatoid diseases, Hepatitis, HIV, corticosteroid therapy and chemotherapy.

Laboratory parameters such as leukocyte count, platelet count, hemoglobin levels, C-reactive protein (CRP), and creatinine were analyzed. Laboratory values were obtained preoperatively on the day of or before surgery and the highest value was recorded postoperatively within the first 5 days after surgery. Pus from the intracerebral abscess was examined through microscopy, Gram staining, and polymerase chain reaction (PCR) to identify microbial pathogens.

Data on the length of intensive care unit (ICU) stay and the need for additional neurosurgical interventions were also recorded.

Imaging analyses included the anatomical location of the abscess, its distance from the ventricles and cortex, the number of lesions, abscess volume (in milliliters), perilesional edema volume (in milliliters), and the maximal midline shift (in millimeters) (Fig. 1). Volumetric analysis was performed using inomed IPS 5 software (inomed GmbH, Emmendingen, Germany). All imaging analyses were done independently by two observers and to ensure interobserver reliability. The analysis was then made by taking the average of their results.

### Statistical analysis

Statistical analysis was performed using SPSS (IBM Corp. Released 2020. IBM SPSS Statistics for Windows, Version 27.0. Armonk, NY: IBM Corp.).

Continuous variables are presented as means and standard deviations (± SD) unless declared otherwise. Categorial variables are presented as absolute number (n) and percentage (%). Fisher Exact Tests and Fisher-Freeman-Halton-Tests were applied for categorial variables and Cramer’s V is provided for significant results. We used Mann–Whitney-U-Tests and Kruskal–Wallis-Tests for nonparametric testing of metric variables. Correlation analyses were performed using Spearman’s correlation with Spearman’s Rho (*r*_*s*_) and p-values displayed. A *p*-value < 0.05 was considered significant in two-tailed testing.

A Receiver Operating Characteristic (ROC) analysis was performed to assess the prognostic accuracy of the significant factors. The area under the curve (AUC), 95% confidence intervals (95%CI) and significance are reported here.

### Ethical approval and informed consent

All procedures performed in this study involving human participants were in accordance with the ethical standards of the 1964 Helsinki declaration and its later amendments. Informed consent was waived due to the retrospective study design. The study and that informed consent was waived were approved by the Institutional Review Board, Paracelsus Medical University, Nuremberg, IRB 2024.05.

## Results

### Patient cohort

**Fig. 2:**
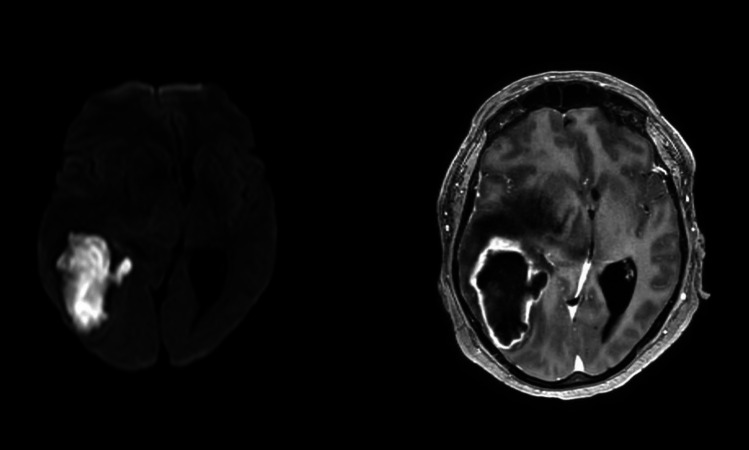
Visualization of an brain abscess by MRI. Diffusion weighted Imaging (left) demonstrating restriction of diffusion, T1 sequence with Gadolinium (right) demonstrating a large abscess with enhancement of the abscess capsule and perilesional edema leading to midline shift

A total of 60 patients (36.7% female) with a mean age of 48.5 ± 20.8 years were included in the analysis. The median CCI was 2 ± 3. Elevated leukocyte count was observed in 35 patients (58.3%), and elevated CRP levels were present in 41 patients (68.3%). Streptococci were the most commonly isolated pathogens (38.3%). The most frequent location of the brain abscesses was the frontal lobes (38.3%), and 56 patients (93.3%) had a single lesion. The mean abscess volume was 18.3 ± 20.3 mL, with a corresponding perilesional edema volume of 52.7 ± 40.4 mL (Fig. 2). 6 patients (10%) suffered from potential immune deficiencies, 3 patients with rheumatoid arthritis, 2 patients with hepatitis and 1 patient receiving corticosteroids.

Baseline data are summarized in Table 1.

**Table 1:** Baseline data of the patient cohort

Item	Mean (SD)/ Median (IQR)*	N (%)
Age	48.5 (20.8)	
Female		22 (36.7)
Focal deficit		29 (48.3)
DOC		12 (20)
Preoperative mRS ≥ 3		18 (30)
Postoperative mRS ≥ 3		6 (10)
Charlson Comorbidity Index	2 (3)	
Immunodeficiencies		
Rheumatoid arthritis Hepatitis Corticosteroids		3 (5) 2 (33.3) 1 (16.7)
Source of abscess		
Local spread Dental origin Systemic origin Entrance through skin lesions/wounds Other		11 (18.3) 20 (33.3) 0 (0) 1 (1) 3 (5)
Abscess volume (ml)	18.3 (20.3)	
Edema volume (ml)	52.7 (40.4)	
Midline shift (mm)	2.8 (4.0)	
Abscess location		
Frontal		23 (38.3)
Temporal		9 (15)
Parietal		14 (23.3)
Occipital		5 (8.3)
Cerebellar		4 (6.7)
Basal ganglia and insula		4 (6.7)
Pituitary gland		1 (1.7)
Single lesion		56 (93.3)
Microsurgery		22 (36.7)
Need for additional neurosurgical procedure		20 (33.3)
Isolated pathogen		
Streptococci		23 (38.3)
Fusobacterium spp.		6 (10)
Mixed infection		18 (30)
Others		6 (10)
Isolated pathogen according to Gram staining		
Gram positive		26 (43.3)
Gram negative		11 (18.3)
Gram positive and Gram negative		16 (26.7)
No isolated pathogen		7 (11.7)
Elevated CRP	41 (31.7)	
Leucocyte count (/nl)	11.7 (4.5)	

*Was used to present CCI

### Risk factors for poor postoperative status

In the postoperative course, 6 patients (10%) were classified with an mRS score of ≥ 3, including 2 patients (3.3%) who died during their hospital stay. Notably, 45 patients (75%) showed an improvement of at least one point on the mRS. Preoperative DOC (*p* = 0.012) and elevated preoperative CRP levels (*p* = 0.002) were identified as risk factors for an unfavorable outcome. ROC analysis showed a good prognostic accuracy of both AUCs with 0.8 (CI = 0.63–0.97, *p* = < 0.001) for elevated CRP levels and 0.73 (CI = 0.46–1.0, *p* = 0.042) for DOC. The cut-off value for CRP was 1.35 mg/dl (Sensitivity 1.0, Specificity 0.43). Patients with poor outcomes had a significantly longer ICU stay (*p* = 0.001). However, poor preoperative status was not necessarily associated with a poor postoperative outcome (*p* = 0.06). Furthermore, neither edema volume (*p* = 0.93) nor the CCI (*p* = 0.3) were significantly associated with poor outcomes. While larger abscess volume (37.4 mL vs. 16.1 mL, *p* = 0.065) and a shorter mean distance to the ventricles (3 mm vs. 11.42 mm, *p* = 0.086) trended toward significance, there was no correlation between distance to the cortex and outcome (*p* = 0.99) or the isolated pathogen (*p* = 0.81). Both, Immunodeficiency (*p* = 0.73) and presumed source of abscess (*p* = 0.33) did not impact the postoperative status. Data is presented in Table 2.

**Table 2:** Radiographic and clinical parameters in patients with good and poor postoperative outcome

Item	Postoperative mRS ≥ 3		Postoperative mRS < 3		
	Mean (SD)/Median (IQR)*	N (%)	Mean (SD)/Median (IQR)*	N (%)	p
Age	52.2 (27.9)		48.0 (20.2)		0.51
Female		2 (33.3)		22 (40.7)	1.0
Focal deficit		1 (16.7)		5 (9.3)	0.2
DOC		4 (66.7)		2 (3.7)	**0.012**
Charlson Comorbidity Index	2 (6)		1 (3)		0.3
Abscess volume (cm^3^)	37.4 (44.5)		16.1 (15.0)		0.065
Edema volume (cm^3^)	64.7 (60.4)		51.3 (38.1)		0.93
Midline shift (mm)	5.7 (6.8)		2.4 (3.6)		0.34
Abscess location					0.51
Frontal		1 (16.7)		22 (40.7)	
Temporal		2 (33.3)		7 (13.0)	
Parietal		2 (33.3)		12 (22.2)	
Occipital		0		5 (9.3)	
Cerebellar		1 (16.7)		3 (5.6)	
Basal ganglia and insula		0		4 (7.4)	
Pituitary gland		0		1 (1.9)	
Single lesion		6 (100)		50 (92.6)	1.0
Microsurgery		2 (33.3)		4 (7.4)	1.0
Need for additional neurosurgical procedure		3 (50)		17 (31.5)	0.39
Duration ICU (days)	17.2 (17.6)		3.4 (5.6)		** < 0.001**
Inpatient stay (days)	17.5 (10.0)		25.4 (12.1)		0.12
Isolated pathogen					0.55
Streptococci		4 (66.7)		19 (35.2)	
Fusobacterium spp.		1 (16.7)		5 (9.3)	
Mixed infection		1 (16.7)		17 (31.5)	
Others		0		6 (11.1)	
Isolated pathogen according to Gram staining					0.81
Gram positive		4 (66.7)		22 (40.7)	
Gram negative		1 (16.7)		10 (18.5)	
Gram positive and Gram negative		1 (16.7)		15 (27.8)	
No isolated pathogen		0		7 (13.0)	1.0
CRP (mg/dl)	10.7 (9.9)		1.8 (3.9)		**0.002**
Leucocyte count (/nl)	13.4 (6.4)		11.5 (4.3)		0.66

*Was used to present CCI

### Risk factors for poor preoperative status

Preoperatively, 18 patients (30%) had an mRS score of ≥ 3. Twenty-nine patients (48.3%) presented with focal neurologic deficits, and 12 patients (20%) had DOC. Patients with poor preoperative status had significantly higher leukocyte counts (*p* = 0.007), while CRP levels were elevated as well, though not significantly (*p* = 0.39). ROC analysis showed good prognostic accuracy of elevated leucocyte levels with an AUC of 0.84 (CI = 0.71–0.98, *p* = < 0.001). We identified a cut-off for leukocyte count of 10.9/nl (Sensitivity 0.83, Specificity 0.41). Radiographic parameters, the isolated pathogen (*p* = 0.1), immune deficiencies (*p* = 0.85) and the presumed source of the abscess (*p* = 0.25) did not influence the preoperative status (Table 3).

**Table 3:** Radiographic and clinical parameters in patients with good and poor preoperative status

Item	Preoperative mRS ≥ 3		Preoperative mRS < 3		
	Mean (SD)/ Median (IQR)*	N (%)	Mean (SD)/ Median (IQR)*	N (%)	p
Age	42 (22.2)		51.2 (19.8)		0.11
Female		6 (33.3)		16 (38.1)	0.78
Focal deficit		6 (33.3)		23 (54.8)	0.16
DOC		12 (66.7)		0	** < 0.001**
Postoperative mRS ≥ 3		4 (22.2)		2 (4.8)	0.06
Charlson Comorbidity Index	1 (2)		1 (3)		0.82
Abscess volume (ml)	25.7 (30.9)		15.1 (12.7)		0.34
Edema volume (ml)	58.7 (49.4)		50.1 (36.2)		0.85
Midline shift (mm)	4 (5.3)		2.2 (3.3)		0.38
Abscess location					0.83
Frontal		6 (33.3)		17 (40.5)	
Temporal		5 (27.8)		4 (9.5)	
Parietal		4 (22.2)		10 (23.8)	
Occipital		1 (5.6)		4 (9.5)	
Cerebellar		1 (5.6)		3 (7.1)	
Basal ganglia and insula		1 (5.6)		3 (7.1)	
Pituitary gland		0		1 (2.4)	
Single lesion		17 (94.4)		39 (92.9)	1.0
Microsurgery		6 (33.3)		16 (38.1)	0.78
Need for additional neurosurgical procedure		8 (44.4)		12 (28.6)	0.37
Duration ICU (days)	9.2 (13.8)		2.8 (3.5)		**0.035**
Inpatient stay (days)	24.4 (13.7)		26.6 (11.4)		0.74
Isolated pathogen					0.37
Streptococci		7 (38.9)		16 (38.1)	
Fusobacterium spp.		3 (16.7)		3 (7.1)	
Mixed infection		6 (33.3)		12 (28.6)	
Others		2 (11.1)		6 (14.3)	
Isolated pathogen according to Gram staining					0.1
Gram positive		7 (38.9)		19 (45.2)	
Gram negative		6 (33.3)		11 (26.2)	
Gram positive and Gram negative		5 (27.8)		5 (11.9)	
No isolated pathogen		0		7 (16.7)	0.091
CRP (mg/dl)	5.4 (8.6)		1.6 (2.7)		0.39
Leucocyte count (/nl)	14.7 (5.9)		10.4 (3.1)		**0.007**

*Was used to present CCI

Age and comorbidities were well-balanced between patients with good and poor preoperative status. A pathogen was isolated from the abscess in all patients with an mRS score of ≥ 3, while isolation was not possible in 7 patients with an mRS score < 3 (*p* = 0.1). Data are presented in Table 2.

### Surgical technique

A total of 22 patients (36.7%) underwent open surgery, while 38 (63.3%) received stereotactic or navigated aspiration. An intralesional drain was used in 32 procedures (53.3%). All basal ganglia and insula lesions were treated stereotactically. These lesions were generally smaller than those treated microsurgically, though the size difference was borderline significant (*p* = 0.051).

Twenty patients (33.3%) required at least one additional neurosurgical procedure, but this was not associated with the initial technique (*p* = 0.09) or drain use (*p* = 0.79). Patients needing revision surgery had significantly longer ICU stays (*p* = 0.036). Abscess volume did not significantly differ between treatment groups (*p* = 0.07), and further surgical treatment was not linked to poor postoperative outcomes (*p* = 0.39).

### Follow-up course

Of the 60 patients, 38 attended the 3-month follow-up. Among them, 13 (34.2%) showed further improvement in their mRS score, 20 (52.6%) remained unchanged, and 5 (13.2%) experienced deterioration (Table 4). Only in two of these cases, the decline was due to abscess recurrence; in the remaining three, it was attributed to pre-existing underlying conditions.

**Table 4:** mRS score at 3-month follow-up compared to mRS at discharge

Course	n (%)
Further improvement	13 (34.2)
No change	20 (52.6)
Worsening	5 (13.2)

Overall, our study showed a consistent improvement in patient status as measured by the mRS during the course of their treatment. Starting with a preoperative median mRS of 2, the median improved to 1 at discharge and further to 0 at the 3-month follow-up (Table 5).

**Table 5:** Patient status as measured by the mRS during the course of treatment

mRS	Median (± IQR)
At admission	2 (2)
At discharge	1 (1.75)
3-months follow-up	0 (1)

## Discussion

In our study, six patients had a postoperative mRS score of ≥ 3, with two of them dying. We identified preoperative DOC and elevated CRP levels as risk factors for an unfavorable postoperative outcome while an elevated leukocyte count was associated with poor preoperative status.

### Postoperative outcome

Despite a decrease in mortality rates over the past decades, mortality remains high, with reported rates ranging from 4.9% to 8.5% in other studies [1, 3, 5, 9, 10, 20, 22].

In our study, we report a mortality rate of only 3.3%. This may be due to our shorter postoperative follow-up compared to other studies [1, 10, 13]. Additionally, many studies report outcomes from a mixed population, including patients who received conservative treatment only.

In our study, an unfavorable outcome at discharge, defined as an mRS ≥ 3, was observed in 10% of patients. Xiao et al. reported a significantly higher rate of unfavorable outcomes (38%) with a similar definition, though this was from a mixed population that included a subgroup receiving only conservative treatment. Among the surgery subgroup in their study, the unfavorable outcome rate was 28%, highlighting the efficacy of surgical treatment [8, 23].

Other studies, using a similar definition of unfavorable outcome (dependence in daily life), reported rates between 22% and 25.2% [12, 13, 22].

The differences in unfavorable outcome rates are likely due to variations in the timing of outcome assessment and the heterogeneity of the study populations, particularly in terms of etiology and treatment approach for brain abscesses.

We identified two parameters associated with poor clinical outcomes: preoperative DOC and elevated preoperative CRP levels, both of which were risk factors for an unfavorable outcome.

Preoperative DOC has previously been reported as a negative predictor for outcomes in other studies [10, 11, 17, 23]. A more severe intracerebral infection may contribute to a worse clinical status, from which patients recover poorly. While one might expect greater midline shift or abscess volume to impact outcomes due to increased pus or edema, these factors were not statistically significant in our study, although abscess volume trended higher in patients with poor outcomes. The literature on radiographic factors is conflicting. Demir et al. proposed an “imaging severity index” for brain abscesses, which includes the number and size of the abscess, corresponding edema, and midline shift [10]. However, other studies have concluded that abscess diameter does not significantly influence outcome [23].

CRP may reflect a more severe systemic infection, potentially leading to worse outcomes. In contrast, initial CRP levels did not influence outcomes in the study by Xiao et al. [23].

The prolonged ICU stay in patients with unfavorable outcomes is likely a result of their worse clinical status postoperatively, rather than an additional independent risk factor.

It is noteworthy that of the 18 patients with poor preoperative status, only 6 were classified with a poor postoperative outcome, which may highlight the efficacy of our combined treatment approach. Seventy-five percent of our patients improved by at least one point on the mRS scale.

Although age, immunodeficiency, and other comorbidities, such as diabetes mellitus, have been shown to influence outcomes in other studies [4, 13, 23],these factors were not influencing the outcomes in our study.

Ventricular rupture and proximity to the ventricles have been highlighted in the literature as negative predictors, due to the potential for widespread pus dissemination into the cerebrospinal fluid [4, 13, 19, 22]. In our study, the mean distance to the ventricles was smaller in patients with unfavorable outcomes, though it did not reach statistical significance (3 mm vs. 11.42 mm). Additionally, proximity to the cortex and suspected subarachnoid spread of the infection did not influence outcomes in our cohort.

Finally, neither preoperative nor postoperative status correlated with the isolated pathogen.

### Preoperative clinical presentation

A poor preoperative mRS score was associated with higher leukocyte counts, which may reflect a more active systemic infection that contributes to worsening clinical status. Elevated leukocyte counts are not always seen in patients with brain abscesses. In our study, only 58.3% of patients had elevated leukocyte counts, consistent with findings from Huang et al. [12].

DOC was observed in 20% of our study cohort. DOC has been previously reported in patients with brain abscesses and is associated with additional risks, such as aspiration and pneumonia [1, 21].

### Surgery

All patients in this study were treated surgically based on a case-by-case decision made by the surgical team. The surgical approach included either navigated/stereotactic aspiration or open surgical drainage.

In our study, there was no significant difference in clinical outcomes or re-surgery rates between these two subgroups. While surgery is generally indicated for brain abscesses, the optimal surgical approach remains a subject of debate [2, 13]. Previous studies have reported no significant difference in clinical outcomes between these two treatment options after 3 months [13, 20, 24]. However, microsurgical resection has been associated with earlier neurological recovery and fewer instances of re-surgery [14, 17, 20, 24].

To the best of our knowledge, there is no randomized controlled trial comparing the effectiveness of different surgical strategies for brain abscesses. As such, the decision regarding the type of surgery should be made on an individual basis.

### Follow-up course

In our study, the clinical course after three months showed further improvement in the mRS, with most patients experiencing either no symptoms or only minor ones. This highlights the prolonged treatment process, where surgery is followed by weeks of antibiotic therapy before patients reach their pre-abscess condition. Quite interestingly, our patient population consisted of relatively healthy individuals, with few experiencing severe illness or limitations prior to the abscess.

Only two cases of recurrent abscess required re-hospitalization. However, the small sample size limits our ability to identify risk factors for poor outcomes after three months. Overall, the study suggests that most poor outcomes occur during the inpatient stay, while the majority of patients recover after discharge.

### Strengths and limitations

This study is inherently limited by its retrospective design and the short duration of outcome evaluation. Additionally, the small sample size precluded a meaningful multivariate analysis, preventing the identified risk factors from being confirmed as independent predictors.

However, a key strength is the homogeneous, consecutive cohort of patients with primary brain abscesses, all of whom underwent preoperative MRI and received combined treatment (surgery plus anti-infective therapy). Additionally, the use of volumetric measurement for both abscess and edema volume through 3-D reconstruction represents another strength of the study.

## Conclusion

Preoperative DOC and elevated CRP levels were identified as predictors of unfavorable outcomes. Elevated leukocyte count also served as a predictor for poor preoperative status. Patients with poor outcomes had longer ICU stays.

## Data Availability

Data is provided within the manuscript.

## Abbreviations

MRI: Magnetic resonance imaging
mRS: Modified Rankin scale
DOC: Disturbance of consciousness
GCS: Glasgow Coma Scale
ICU: Intensive care unit
CCI: Charlson Comorbidity Index
CRP: C-reactive Protein
ml: Milliliter
mm: Millimeter
SD: Standard deviation
